# Development of a bacterial bioassay for atrazine and cyanuric acid detection

**DOI:** 10.3389/fmicb.2015.00211

**Published:** 2015-03-17

**Authors:** Anna Hua, Hervé Gueuné, Mickaël Cregut, Gérald Thouand, Marie-José Durand

**Affiliations:** ^1^Nantes University, Campus de la Courtaisière - IUT, UMR CNRS 6144 GEPEA, CBACLa Roche-sur-Yon, France; ^2^CORRODYS, Centre de Corrosion Marine et BiologiqueCherbourg, Octeville, France

**Keywords:** bacterial bioelement, bioluminescence, *luxCDABE*, biodetection, biosensor, atrazine, cyanuric acid, bioassay

## Abstract

The *s*-triazine herbicides are compounds which can disseminate into soils and water. Due to their toxic effects on living organisms, their concentrations in drinking water are legislated by WHO recommendations. Here we have developed for the first time, to the best of our knowledge, an alternative method for physicochemical quantification using two bioluminescent bacterial biosensors: *E. coli* SM003 for cyanuric acid detection and *E. coli* SM004 for both atrazine and cyanuric acid detection. The concentration of cyanuric acid detection for *E. coli* SM003 ranges from 7.83 μM to 2.89 mM, and for *E. coli* SM004 ranges from 0.22 to 15 μM. Moreover, atrazine detection by *E. coli* SM004 ranges from 1.08 to 15 μM. According to WHO recommendations, the cyanuric acid detection range is sensitive enough to discriminate between polluted and drinking water. Nevertheless, the detection of atrazine by *E. coli* SM004 is only applicable for high concentrations of contaminants.

## Introduction

The *s*-triazine family herbicide is used for agricultural purposes, such as preventing pre and post-emergence weeds in crops. These herbicides are mobile molecules that disseminate into soils and water. The process leads to the accumulation of a cyanuric acid compound, which is a building block common to the diverse family of *s*-triazine (Ralebitso et al., 2002).

These polluted environments have various toxic effects on all living organisms, from aquatic organisms to animals and human beings. Due to these effects, atrazine use is currently forbidden in most European countries, but still widely used in other parts of the world. According to the WHO, atrazine and cyanuric acid concentrations in drinking water are limited to 2 μg.L^−1^ (0.01 μM) and 40 mg.L^−1^ (310 μM), respectively (World Health Organization, 2011).

Monitoring atrazine in environments can be performed by various chemical analyses, including gas chromatography coupled with physico-chemical detection systems. Even if these methods are sensitive, they involve organic extraction of the sample prior to proper analysis, which does not reflect the bioavailable fraction of the contaminant.

In this study, we propose for the first time an alternative method for cyanuric acid and atrazine detection using bacterial biosensors technology. The adopted strategy for construction of the bioelements is uncommon because it integrates, in addition to inducible detection of cyanuric acid, atrazine biodegradation pathway.

**Figure 1:**
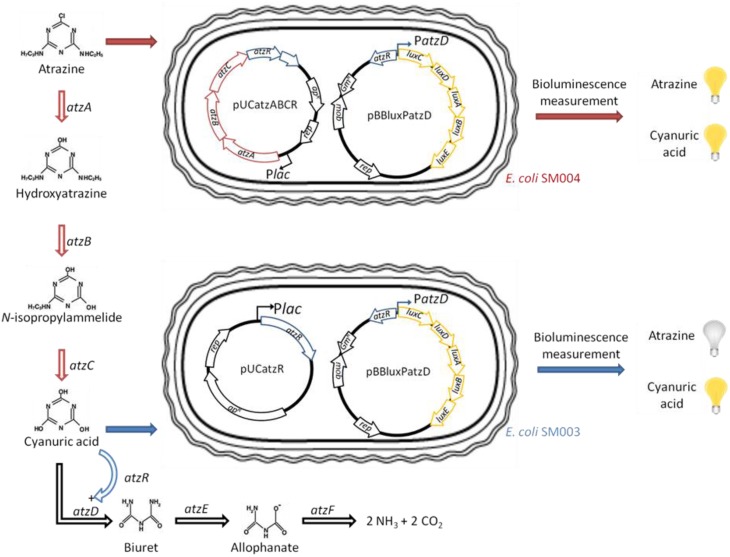
**Atrazine degradation pathway and strategy for atrazine and cyanuric acid detection**. In blue are the elements necessary for cyanuric acid detection. In red are the elements necessary for atrazine degradation into cyanuric acid.

To address this goal, the pADP-1 plasmid, borne by *Pseudomonas* sp. pADP-1 and well-studied for its ability to degrade atrazine, was used for genetic construction (Mandelbaum et al., 1995) (Figure 1). Atrazine biodegradation pathway is encoded through the *atz* genes, which can be divided into two sets: (*i*) the constitutive *atzA*, *atzB*, and *atzC* genes responsible of atrazine degradation into cyanuric acid and (*ii*) the inducible *atzDEF* operon, leading to cyanuric acid mineralization into NH_3_ and CO_2_. Moreover, the *atzDEF* expression requires the constitutive expression of its regulator *atzR* (Govantes et al., 2010).

Since atrazine degradation genes into cyanuric acid are constitutively expressed, their promoters cannot be used as bioreporters. Therefore, atrazine biodetection can be developed solely after its initial biodegradation into cyanuric acid. In these conditions, we developed a two-step strategy consisting in developing two complementary strains: one for cyanuric acid detection only and the second for atrazine biodetection after its biodegradation into cyanuric acid (Figure 1).

## Materials and methods

### Bacterial strains, media, and growth conditions

Bacterial strains (Table 1) were grown in Terrific Broth (TB) medium (Sigma-Aldrich, Fr) at 37°C in the presence of ampicillin (100 mg.L^−1^, Sigma-Aldrich, Fr) and gentamicin (25 mg.L^−1^, Sigma-Aldrich, Fr), and agitated at 250 rpm in baffled shack flasks.

**Table 1:** **Bacterial strains and plasmids used for biosensor construction**.

**Name**	**Description**	**References**
**BACTERIAL STRAINS**		
*E. coli* JM109	*endA1 glnV44 thi-1 relA1 gyrA96 recA1 mcrB+ Δ(lac-proAB) e14- [F′ traD36 proAB+ lacIq lacZΔM15] hsdR17(rK-mK+)*	Sigma-Aldrich
*Pseudomonas* sp. ADP	pADP-1, Hg^R^	Mandelbaum et al., 1995
*E. coli* SM003	*E. coli* JM109, pBBluxatzD, pUCatzR, Ap^R^, Gm^R^	This study
*E. coli* SM004	*E. coli* JM109, pBBluxatzD, pUCatzABCR, Ap^R^, Gm^R^	This study
**PLASMIDS**		
pBfiluxCDABE	Ap^R^, *ptac::luxCDABE*, constitutive expression of the *Aliivibrio fischeri* bioluminescence *luxCDABE* operon	Charrier et al., 2011
pBfiluxMCS	Ap^R^, pBfiluxCDABE containing MCS and *luxCDABE* operon	This study
pBBR1MCS-5	Gm^R^, cloning vector rep, *lacZ*'	NCCB 3437
pBBluxMCS	Gm^R^ pBBR1MCS-5 containing MCS and *luxCDABE* operon	This study
pBBluxPatzD	Gm^R^ pBBluxMCS containing *atzR* and PatzDEF promoter	This study
pUC19	Ap^R^, high copy number cloning vector	Invitrogen
pUCatzR	Ap^R^ pUC19 containing *atzR* gene	This study
pUCatzABC	Ap^R^ pUC19 containing *atzA*, *atzB* et *atzC* genes	This study
pUCatzABCR	Ap^R^ pUCatzR containing *atzA*, *atzB* et *atzC* genes	This study

### Pollutant solutions

Stock solutions of cyanuric acid and atrazine (both from Sigma-Aldrich, Fr, purity ≥98%) were made in isopropanol/ultrapure water (20/80 v/v) at concentrations of 2.5 mM and 150 μM, respectively. These solutions were stocked at −20°C for 3 months and diluted in ultrapure water to required concentrations prior to use. Because solubility thresholds differ from cyanuric acid and atrazine (21 mM and 161 μM, respectively), the concentration ranges used for exposure were between 4 nM and 4 mM and between 8 nM and 15 μM, respectively.

### Molecular biology for reporter plasmid construction

In this study, we aimed to develop a set of two complementary strains reporting cyanuric acid and atrazine. The strategy employed was based on the use of the molecular resources of the pADP1 plasmid, bearing the genes encoding for (*i*) the biodegradation of atrazine into cyanuric acid, encoded by the *atzA*, *atzB*, and *atzC* genes; and (*ii*) cyanuric acid biosensing achieved by the *atzD* promoter and its regulator *atzR. Lux CDABE* reporter genes were used from *Allivibrio fischeri* (Supplementary Data).

Once constructed, *E. coli* JM109 cells (Sigma-Aldrich, Fr) were transformed by pBBluxPatzD and pUCatzR for *E. coli* SM003 and pBBluxPatzD and pUCatzABCR for *E. coli* SM004 (Charrier et al., 2011). Those transformations lead to the bioelements for (*i*) cyanuric acid and (*ii*) both atrazine and cyanuric acid biodetection, respectively (Table 1).

### Bioluminescence measurement and statistical analysis

After overnight growth at 37°C in TB medium agitated at 250 rpm, microbial cells were diluted to an absorbance (A_620_) of 0.2 and cultivated at 30°C to promote luciferase protein expression. When biomass reached A_620_ = 0.7, IPTG induction (2 mM) was performed to induce AtzABCR biosynthesis. After an average of three bacterial generations (around A_620_ = 1.7), bacterial culture was diluted at A_620_ = 0.25 in 1/10 TB medium without antibiotics, to obtain 2 mL of bacterial suspension. The latter was then induced with various amounts of pollutants (cyanuric acid or atrazine) and shaken in a 24-well microplate for five minutes to enable the homogenization of the chemical in the media. Finally, 200 μL of the bacterial suspension was transferred into a white 96-well microplate for bioluminescence measurement. Bioluminescence was recorded over 4 h with an acquisition time of 1 second per well at 30°C using a luminometer (Berthold, Fr).

Raw bioluminescence results were represented as Relative Luminescence Units per second (RLU.s^−1^). The Induction Ratio (IR) was calculated as follows: IR = (RLU.s^−1^)_I_/(RLU.s^−1^)_0_, where (RLU.s^−1^)_I_ is bioluminescence after exposure with the pollutant, and (RLU.s^−1^)_0_ is the bioluminescence of the bacterium in the absence of pollutant.

Statistical analyses were performed with GraphPad Prism software. Because of the many dose-response curves fit to a sigmoid, the logistic curve log(substance) vs. response model was applied. This model allows for the calculation of the bioluminescence value Y in presence of the pollutant at the concentration X. This equation takes into account four parameters: (*i*) the saturation signal corresponding to the maximum IR value; (*ii*) the background signal corresponding to the minimum IR value; (*iii*) the log(IR_50_) that provokes a response halfway between saturation and background signals and (*iv*) the Hill slope describing how steep the curve is. The modeled curve is defined as follows:

$$\begin{array}{l} \text{Bioluminescence Y}=\text{Background}+\\ \;\frac{\left(\text{Saturation}-\text{Background}\right)}{1+10^{[(\text{log}(\text{IR}50)-\text{log}(\text{X})]\text{ x Hill slope}}} \end{array}$$

Moreover, the following equation allows for the calculation of detection and saturation limits, with F = 10 and F = 90, respectively.

$$\text{log}({\text{IR}}_{\text{F}})\;=\;\log{\text{IR}}_{50}+\frac{1}{\text{Hill slope x log}\left(\frac{\text{F}}{100-\text{F}}\right)}$$

## Results and discussion

### Cyanuric acid biodetection by *E. coli* SM003 and *E. coli* SM004

**Figure 2:**
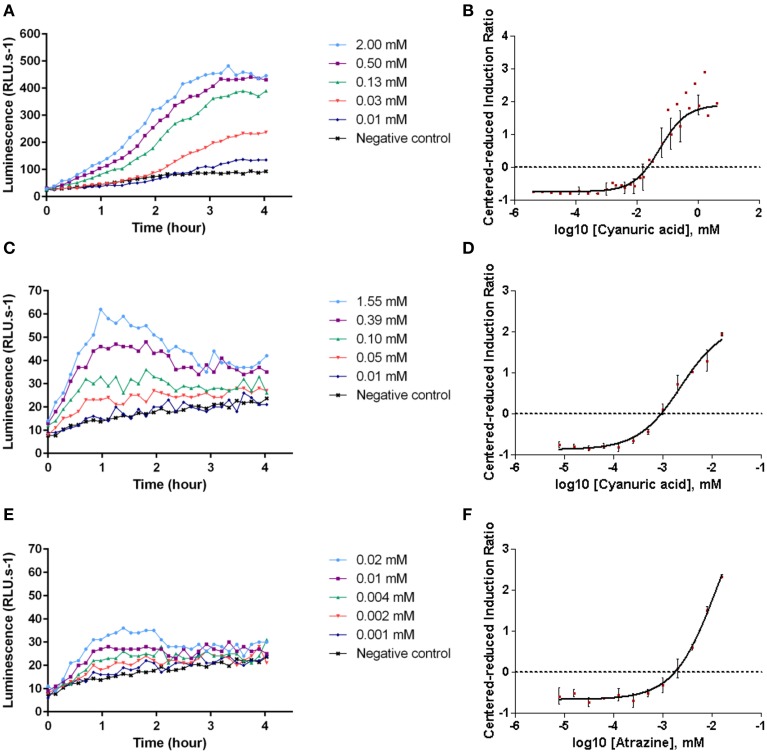
**Example of the kinetic of bacterial bioluminescence production**. *E. coli* SM003 strain in the presence of cyanuric acid **(A)**, *E. coli* SM004 strain in the presence of cyanuric acid **(C)** or atrazine **(E)**. Modeled curves of centered-reduced induction ratio for *E. coli* SM003 at an exposure time of 2 h 30 min in the presence of cyanuric acid **(B)**, and *E. coli* SM004 at an exposure time of 1 h in the presence of cyanuric acid **(D)** or atrazine **(F)**. Bioluminescence measurements and induction ratio are calculated from duplicate of three independent experiments.

*E. coli* SM003 strain was assessed for cyanuric acid biodetection. In the absence of the pollutant, *luxCDABE* genes expression were low and a basal bioluminescence was measured (approximately 70 RLU.s^−1^). In the presence of increasing concentrations of cyanuric acid, bioluminescence increased (Figure 2A). Thus, in the range of tested concentrations of cyanuric acid, bacterial response was concentration-dependent, demonstrating that the *E. coli* SM003 strain can be used as a cyanuric acid bioreporter, with a detection limit of 7.82 μM.

Bioluminescence levels varied over time, increasing until a maximum and then slightly decreasing. This can be explained by the metabolic limitations of the bacterium, which possesses a defined luciferase substrate turnover and so, a limited ability to cope with a defined amount of pollutants. Moreover, background bioluminescence depended on the experiment (68 ± 26 RLU.s^−1^ for *E. coli* SM003 in three replicates). Thus, bioluminescence variations intra- and inter-experimentally can be explained by differences in bacterial physiological states.

To take into account bioluminescence level variations, the time after exposure for IR calculation was fixed. Based on the higher value of the population variance, which reflects the broader spread of bioluminescence values at a given time, the optimal time for bioluminescence analysis was set at 2 h 30 min after cyanuric acid exposure.

A predictive model was established to represent bacterial bioluminescence as a function of pollutant concentrations. The r^2^ value of the fitted curve was approximately 0.96, indicating that the applied model was valid (Table 2). The modeled sigmoid presented a bottom and a top plateau (Figure 2B), which can be defined as detection and saturation thresholds. By establishing them as IR_10_ and IR_90_, respectively, cyanuric acid detection by the *E. coli* SM003 biosensor ranges between 7.83 μM to 2.89 mM. The range of detected concentrations is wide enough to discriminate between drinking and polluted water according to WHO recommendations (310 μM of cyanuric acid in drinking water). This range can also be able to detect significant pollution because cyanuric acid solubility is defined as 2.7 g.L^−1^ (21 mM), proving that all the substance is bioavailable.

**Table 2:** **Modeled curve parameters**.

**Pollutant**	**Bacterial strain**	**Modeled curve equation**	***r*^2^**	**Detection limit**	**Saturation limit**
Cyanuric acid	*E. coli* SM003	$\text{Y}=-0.7117+\frac{\left(2.027+0.7117\right)}{1+10^{[\left(-1.325\;-\text{ log}(\text{X}\right)]\text{ x }1.341}}$	0.9577	7.83 μM	2.89 mM
	*E. coli* SM004	$\text{Y}=-0.8613+\frac{\left(2.101+0.8613\right)}{1+10^{[-2.672\;-\text{ log}(\text{X})]\text{ x}1.070}}$	0.9871	0.22 μM	15 μM
Atrazine	*E. coli* SM004	$\text{Y}=-0.6333+\frac{\left(3.508+0.6333\right)}{1+10^{\left[-2.128\;-\text{ log}\left(\text{X}\right)\right]}\text{ x}1.252}$	0.9952	1.08 μM	15 μM

*The modeled curve equation is based upon the equation described in the Materials and Methods section. The detection and saturation limits are defined as log(IR_10_) and log(IR_90_), respectively*.

Upon cyanuric acid exposure, *E. coli* SM004 bioluminescence levels increased with the presence of increased amounts of pollutant in a dose-dependent manner. Based on the population variance study, the optimal bioluminescence analysis time was set as 1 h after pollutant exposure (Figure 2C). The applied model (Figure 2D and Table 2) defined the detected range of cyanuric acid as ranging from 0.22 to 15 μM. Thus, the range of detected concentrations of cyanuric acid is sensitive enough to discriminate between drinking and polluted water according to WHO recommendations.

Even when *E. coli* SM003 and *E. coli* SM004 bioelements were both designed for cyanuric acid detection, differences were shown. In comparison with *E. coli* SM003, the *E. coli* SM004 growth rate is reduced (0.82 vs. 0.49 h^−1^ in TB medium at 30°C), bioluminescence levels are lower and the cyanuric detection range is lower. These phenomena could be explained by the presence of the three *atzA*, *atzB*, and *atzC* supplemental genes in the *E. coli* SM004 bioelement, which increases energy requirements. It could be supposed that *E. coli* SM004 has less energy to cope with the pollutant so, small amounts of pollutant will have larger effects and lead to more sensitive detection with a lower range.

### Atrazine biodetection by *E. coli* SM004

*E. coli* SM004 bioluminescence production in the presence of atrazine was concentration-dependent (Figure 2E). Based on the modeled sigmoidal curve, detectable atrazine concentrations were comprised between 1.08 and 15 μM (Figure 2F and Table 2). The detection limit is not as low as required to detect non-drinkable water, but is sufficient to detect high levels of atrazine contamination if atrazine solubility is defined at 34.7 mg.L^−1^ (161 μM).

In *E. coli* SM004, the less sensitive detection of atrazine in comparison with cyanuric acid can be explained by the presence of the three supplemental genes. They imply the launching of an enzymatic machinery to achieve the production of cyanuric acid before its bioreporting by another enzymatic system, which also requires more energy. More generally, this biosensor is limited by the ability to manage the presence of atrazine degradation genes and their related metabolic load.

Moreover, it was shown that in the presence of different concentrations of atrazine the bioluminescence produced by *E. coli* SM003 was not different from the background value (data not shown).

## Conclusion

Many bioluminescent engineered bacteria have been reported in the literature, most of them dedicated to metals detection (Sorensen et al., 2006; Magrisso et al., 2008; Van der Meer and Belkin, 2010; Eltzov and Marks, 2011, for review). Xu et al. (2014) recently reviewed whole- cell bioluminescent bioassay for organic compounds detection, nevertheless none of them are dedicated to triazine.

In this study, we constructed two bacterial bioelements for pollution assessment by the *s*-triazine family compound: one for cyanuric acid, and one for both cyanuric acid and atrazine detection. *In vitro* studies have shown that, individually, those bioelements are able to detect and quantify pollutants by luminescence measurement.

With environmental samples, the detection of pollutants and discrimination between atrazine or cyanuric acid compounds can be performed by coupling the two bioelements. Because the cyanuric acid detection range differs from *E. coli* SM003 and *E. coli* SM004, the detection range of multiple biosensors corresponds to the detection range of the more sensitive strain, i.e., *E. coli* SM004 (0.22–15 μM). This strain is sensitive enough to discriminate cyanuric acid pollution in drinking water, according to WHO recommendations, which limits its concentration at 310 μM.

Atrazine detection limit by *E. coli* SM004 bioelement starts from 1 μM, which allows the detection of large atrazine contaminations only. Non-sensitive detection of atrazine by the *E. coli* SM004 bioelement can be explained by its large energy requirements, due to the presence of the supplemental genes responsible for atrazine degradation into cyanuric acid. Despite the fact that atrazine detection remains to be improved, *s*-triazine family contaminations can easily be detected by the sole detection of cyanuric acid. Indeed, because cyanuric acid is produced following the degradation of the *s*-triazine family, its quantification by the *E. coli* SM004 bioelement is sufficient to discriminate between polluted and drinking water.

Further studies can be conducted to decrease detection limits. It would be interesting to enhance bioluminescence levels by the addition of luciferase cofactors, such as riboflavin. Moreover, an integration of the constitutively expressed *atzABCR* genes into the bacterial genome would reduce the metabolic load due to plasmid replication, remove an antibiotic resistance gene and, finally, simplify the induction protocol without the IPTG addition step. Once these optimization steps are conducted, the biosensors will be validated for cyanuric acid and atrazine detection first on mixtures with different proportions, and then in environmental and complex samples.

### Conflict of interest statement

The authors declare that the research was conducted in the absence of any commercial or financial relationships that could be construed as a potential conflict of interest.
