# Supplementary material for: Development of a bacterial bioassay for atrazine and cyanuric acid detection
Source: Front Microbiol. 2015 Mar 17;6:211. doi: 10.3389/fmicb.2015.00211 (PMC4362333; doi:10.3389/fmicb.2015.00211)
Supplement: Supplementary file 1 [file DataSheet1.DOCX]

**Supplementary Material for
Development of a bacterial bioassay for atrazine and cyanuric acid detection**

**Anna Hua^1^, Hervé Gueuné^1,2^, Mickaël Cregut^1^, Gérald Thouand^1^, Marie-José Durand^1^***

^1^Nantes University, Campus de la Courtaisière - IUT, UMR CNRS 6144 GEPEA, CBAC,

18 Bd Gaston Deferre,

85035 La Roche-sur-Yon cedex, France

^2^CORRODYS, Centre de corrosion marine et biologique, Cherbourg, Octeville-France

**Molecular biology for reporter plasmid construction**

All *atz* genes from *Pseudomonas sp ADP* were amplified from the pADP-1 plasmid by Polymerase Chain Reaction according to standard methods (Supplementary data S1). The genes for cyanuric acid and atrazine bioreporting were inserted in the pBBR1MCS-5 and pUC19 plasmids. These plasmids possess compatible replication origins, which allow them to be maintained in bacterial *E. coli* cells. In pUC19 based plasmids, *atz* genes were under P*lac* control. In *E. coli* JM109 (*lacI* genotype), their expression required an IPTG induction.

S1: Oligonucleotides used for biosensor construction

| **Name** | **Restriction enzymes** | **Annealing temperature (°C)** | **Sequence (5’🡪3’)** |
| --- | --- | --- | --- |
| MCS-F | multiple | 90 | CGAGGATCCGTCGACCCGGGCCATGGCGGCCGCACTAGTTAAGGAGGG |
| MCS-R | multiple | 90 | AATTCCCTCCTTAACTAGTGCGGCCGCCATGGCCCGGGTCGACGGATCCTCG |
| atzD-F | - | 60 | GCGTGCGTCTTGTAGGTGTA |
| atzD-R | *Spe*I | 60 | TTTTTTACTAGTTACGATGTATCACCAGGGCTGT |
| atzR-F | *Pst*I | 67 | AAAAAACTGCAGTAAGGAGGACTCGCATGCGGGCACA |
| atzR-R | *Bam*HI | 67 | AAAAAAGGATCCGTCACGTTGCATTGTGGGTCGT |
| atzA-F | *Hin*dIII | 61 | AAAAAAAAGCTTAAGGAGGAAATTCATGCAAACGCTCAGCATC |
| atz-R | *Spe*I, *Xba*I | 61 | AAAAAATCTAGAACTAGTCCTAGAGGCTGCGCCAAG |
| atzB-F | *Spe*I | 60 | AAAAAAACTAGTTAAGGAGGACCACCATGACCACCACTCTT |
| atzB-R | *Sph*I, *Bam*HI | 60 | AAAAAAGGATCCGCATGCAACTCATCACCGGCCTCA |
| atzC-F | *Sph*I | 55 | AAAAAAGCATGCTAAGGAGGATAAAAATGAGTAAAGATTTTG |
| atzC-R | *Sac*I, *Xho*I | 55 | AAAAAAGAGCTCAAAAACTCGAGTTAGGCAACTATAACCTCATCCT |

**Molecular construction for cyanuric acid and atrazine bioreporting**

The pBBluxPatzD plasmid bears a transcriptional fusion of the *atzD* promoter to the *luxCDABE* operon of *Aliivibrio fischeri.* For this purpose, P*tac* promoter present on the pBfiluxCDABE plasmid [5] was replaced by Multi Cloning Sites (MCS) through NruI and EcoRI double digestion. Fragment containing both MCS and *luxCDABE* operon was isolated and replaced into the pBBR1MCS-5 plasmid by SphI-PstI digestion to achieve pBBluxMCS plasmid production. *atzD* promoter was then inserted into pBBluxMCS upstream *luxCDABE* by BamHI-SpeI double digestion in order to produce the pBBluxPatzD plasmid (Supplementary data S2).


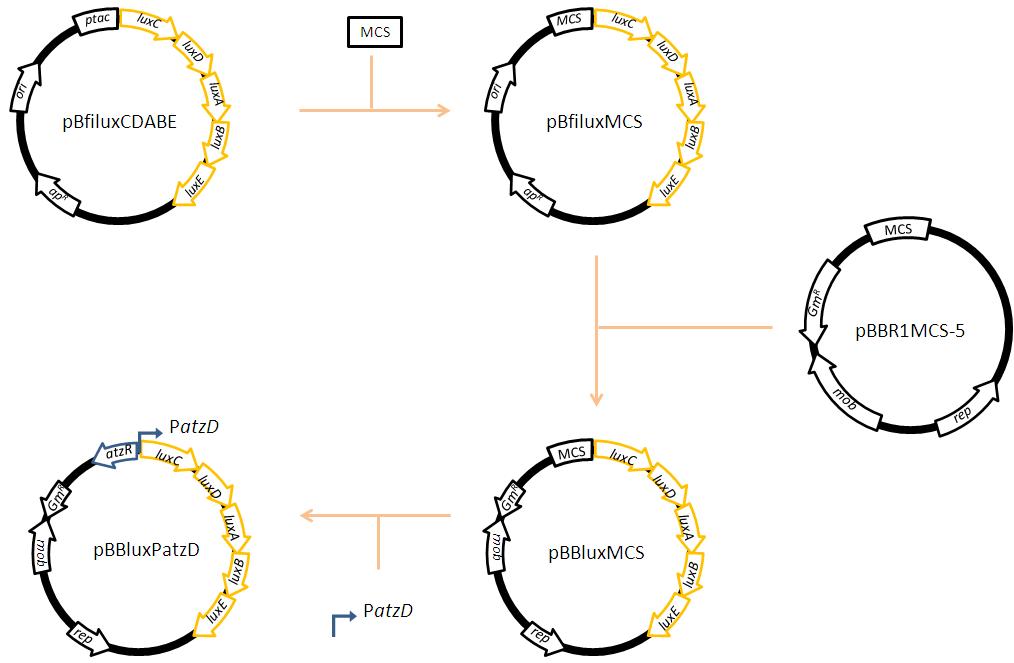


S2: Bacterial plasmid maps and construction strategy for both cyanuric acid and atrazine bioreporting

**Molecular construction specific for cyanuric acid and atrazine detection**

Because P*atzD* requires the constitutive expression of AtzR, the pUCatzR plasmid was constructed. *atzR* was amplified and inserted into pUC19 by BamHI-PstI digestion in order to produce pUCatzR plasmid (Supplementary data S3).

For atrazine degradation into cyanuric acid, the three *atzA*, *atzB* and *atzC* genes were sequentially cloned into pUC19 by sequential double digestion of HindIII-XbaI, SpeI-BamHI and SphI-SacI, respectively, yielding production of the pUCatzABC plasmid. Fragment containing *atzABC* genes was then inserted into pUCatzR plasmid, in order to produce pUCatzABCR plasmid (Supplementary data S3).


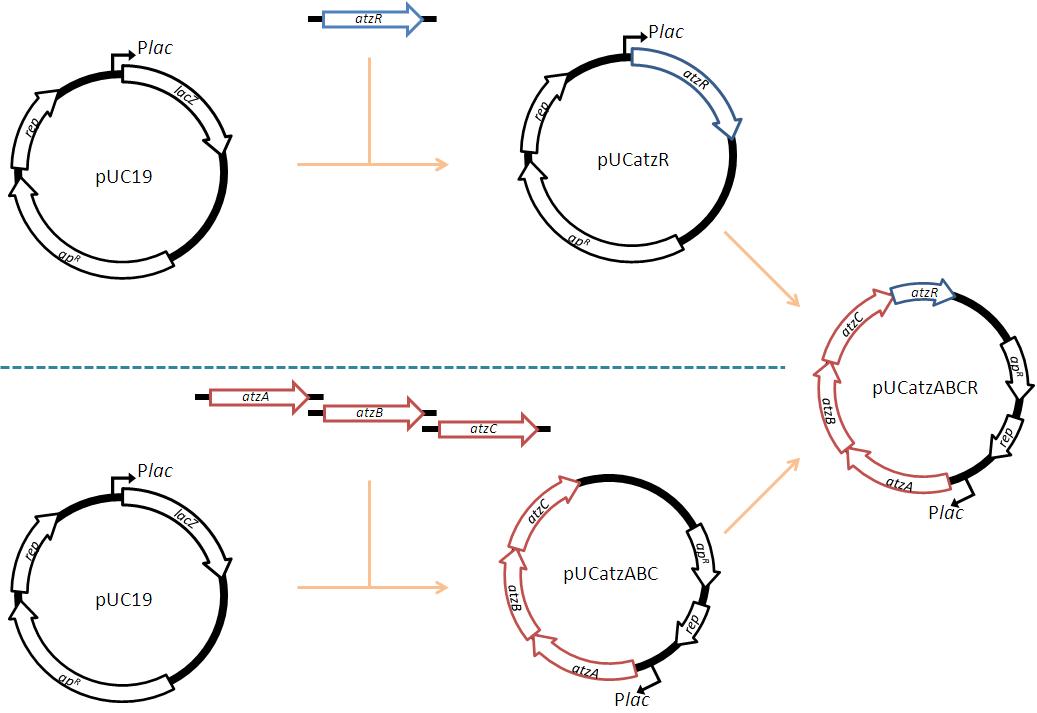


S3: Bacterial plasmid maps and construction strategy for cyanuric acid or atrazine bioreporting.
